# Radiotherapy biobanking: current landscape, opportunities, challenges, and future aspirations

**DOI:** 10.1002/cjp2.246

**Published:** 2021-10-17

**Authors:** Tim H Ward, Duncan C Gilbert, George Higginbotham, Chris M Morris, Valerie Speirs, Nicola J Curtin

**Affiliations:** ^1^ Patient Advocate National Cancer Research Institute (NCRI) London UK; ^2^ Sussex Cancer Centre Royal Sussex County Hospital Brighton UK; ^3^ MRC Clinical Trials Unit at UCL London UK; ^4^ Southmead Hospital Bristol UK; ^5^ Newcastle Brain Tissue Resource, Translational and Clinical Research Institute Newcastle University Newcastle upon Tyne UK; ^6^ Institute of Medical Sciences, School of Medicine, Medical Sciences and Nutrition University of Aberdeen Aberdeen UK; ^7^ Newcastle Centre for Cancer, Translational and Clinical Research Institute, Faculty of Medical Sciences Newcastle University Newcastle upon Tyne UK

**Keywords:** radiotherapy, biobanking

## Abstract

Half of all cancer patients receive radiotherapy, which makes a substantial contribution to their long‐term disease control/cure. There are significant inter‐patient differences in response, both in terms of efficacy and toxicity (frequently delayed onset) which are difficult to predict. With the introduction of technological improvements (e.g. stereotactic body radiotherapy and proton therapy) and development of combination therapies (e.g. radiotherapy and immune checkpoint inhibition), predictive biomarkers are needed even more. Whilst genomic studies have contributed significantly to predictions of response to anticancer therapy, there is no doubt that more information can be gathered from patient tissue samples. Patients are willing to donate their tissues to biobanks and wish them to be used as widely as possible for high‐quality research. We report here a survey of the current practices in the UK from several groups collecting material from patients in radiotherapy trials and have identified barriers to collecting and sharing data and samples. We believe the current situation represents a significant missed opportunity to improve the personalisation of radiotherapy. We propose a greater involvement of patients and/or their advocates, a standardisation of the patient information leaflet, consent form content and data set, with easy linkage to clinical data, which would facilitate widespread sample and data discovery and availability to other researchers. The greater sharing of data and samples, nationally and internationally, would facilitate robust multicentre studies and avoid duplication of effort.

## Introduction

In high‐income countries, 50% of all cancer patients will receive radiotherapy [1] at some point in their treatment pathway, contributing to the curative treatment of 30–40% of patients [2]. Globally, the number of patients requiring radiotherapy increases year on year [3]. Radiotherapy (often with concurrent chemotherapy) is now the primary standard of care treatment for many cancers to facilitate organ preservation (e.g. head and neck, cervical, and anal cancers) with high rates of cure [4, 5]. Radiotherapy remains an alternative to surgery across a number of situations (e.g. prostate and bladder cancers [6, 7]) and as adjuvant therapy in, for example, breast cancer [8]. Technical refinements are facilitating hypo‐fractionated treatments leading ultimately to stereotactic body radiotherapy [9] with patients receiving curative doses of radiotherapy in only a few treatments. Equally, novel immunotherapy combinations (e.g. non‐small cell lung cancer [10]) continue to improve patient outcomes and broaden radiotherapy indications.

However, not all patients are cured and radiotherapy can be associated with serious and delayed sequelae, hence the potential of personalised treatment to improve both treatment efficacy and long‐term quality of life. Progress is slow – for example, although the positive influence of tumour human papillomavirus (HPV) involvement on outcomes after (chemo)radiotherapy for head and neck cancer was first described a decade ago [11], trials of dose modulation based on this biomarker are yet to report [12]. Other studies utilise more general biomarkers of risk [13] as opposed to specific predictors of radiotherapy effect/toxicity.

Preclinical studies have identified multiple determinants of both radio‐sensitivity and radio‐resistance that have the potential to be used as predictive biomarkers for both response and undesirable sequelae. Searchable databases of genomic and transcriptomic information have contributed significantly to biomarker identification, notably The Cancer Genome Atlas [14]. The collection of blood samples for radiogenomics has accelerated recently [15]. However, to fully translate the accumulating knowledge into useable biomarkers for precision oncology, the biobanking of properly curated tumour and normal tissue samples, surplus to diagnostic/clinical need, is essential along with appropriate (anonymised) clinical data.

Tissue samples may be diagnostic biopsies, excess tissue from surgery, or collected as part of a clinical trial where participation mandates blood and/or tissue collection. Paired pre‐ and post‐radiotherapy biopsies are extremely valuable for ‘proof of mechanism’ studies but particularly challenging to collect as the patient must consent to repeated invasive procedures. However, there are clinical indications for repeat biopsies post‐treatment such as biopsy or removal of the primary tumour and secondary recurrence or, for example, sentinel lymph node biopsy in patients undergoing radiotherapy for breast cancer [16, 17, 18]. Whilst primary tumour samples can be difficult to obtain, blood sampling provides opportunities for ‘liquid biopsy’ of both circulating cell‐free tumour DNA (ctDNA), circulating tumour cells (CTCs), and tumour extracellular vesicles pre‐ and post‐radiotherapy [19]. These can be collected serially, avoiding the complications of invasive biopsies especially in hard‐to‐reach or high‐risk tissues such as lung and brain, potentially yielding predictive biomarkers of radiotherapy response.

Patients want their material put to good use with the ultimate goal of improving the outcomes of patients in the future [20, 21]. Quoting our patient representative, the key message here is that *‘Biobanks should not be Safety Deposit boxes accessed by the privileged few but more like open access accounts with stakeholders and others having easy access to deposit and withdraw*’ [22].

Whilst clinical trials may include biobanking of patient material, these studies are usually small and collected for a specific purpose, limiting access. Where biobanking is part of a phase I trial of a novel drug, the availability of samples is less likely to be a major issue but, for a widely used treatment modality like radiotherapy, the desirability of sharing the material is increased. A UK survey of pathology departments identified inconsistency in tissue release for clinical trials, with lack of time and/or resources cited as the biggest barriers [23]. This, together with the lack of a standardised approach to consent, collection, handling, and storage, along with an often variable set of associated patient/clinical data, presents challenges for samples to be shared/pooled in order to obtain meaningful data. The UK National Cancer Research Institute (NCRI) recognised this and, with the goal of improving biobanking in general, established the Confederation of Cancer Biobanks [24] in 2006, and incorporated it into the NCRI Cellular Molecular Pathology (CMPath) initiative in 2016; the objective is to share best practice, to improve the coordination between existing collections, and to raise awareness of existing collections. CMPath worked towards improving and standardising sample collection and reporting by devising a freely available Biobanking Sample Quality Improvement Tool [25] highlighted by the need for sample and data standardisation, especially for biomarker studies [26, 27].

In radiotherapy biobanking, the GENEPI‐ENTB2 project (GENEtic pathways for the Prediction of the effect of Irradiation‐European normal and tumour tissue bank and database) sought to address shortfalls by establishing a database of patient treatment and outcome information, linked to a biobank of tissue samples [28]. This was achieved through the coordination of multiple small biobanks throughout the EU into a ‘virtual EU tissue bank’. The GENEPI‐ENTB2 project documented 12,120 samples from 5,844 radiotherapy patients and 960 healthy volunteers [29]. The final report (2011) [30] highlighted challenges with the IT system and resources required for its management but provided a framework for future biobanking [29, 31].

Learning from the experience of GENEPI‐ENTB2 and CMPath, we sought to review the current UK landscape of radiotherapy‐related biobanking and to propose a system of measures that might enable the effective collection, curation, and sharing of this valuable resource given by patients altruistically to hopefully enable improvements in patient outcomes.

## Public and patient involvement in biobanking

Patient material is a valuable resource, patients consent willingly, but their role in biobanking has historically been passive. Healthcare institutes are beginning to recognise patients as actively involved consumers or collaborators in research [32, 33]. Nevertheless, patients still have no say over the scientific or clinical application of samples they have gifted. Patients have a right to know that their samples are valued, stored, and utilised appropriately, and that what will happen to them at the end of their shelf‐life (including disposal) is clearly stated.

Some well‐resourced UK biobanks, such as the charity‐funded Breast Cancer Now Tissue Bank [34]; Blood Cancer UK CellBank [35]; Pancreatic Cancer Research Fund Tissue Bank [36]; and the part government, part charity‐funded Wales Cancer Bank [37], involve patients in governance of the repository and tissue access committees and some include patients in the overall management of the biobank such that they know what happens to their samples and their contribution to research [33]. If we acknowledge that patients should be fully informed and engaged when consenting to donate, then this also applies when they are asked to comment/decide on the use of such samples [32]. Patient representatives can learn how to navigate the esoteric terminology and concepts through the Science for Advocates course developed by the Independent Cancer Patients' Voice [38].

Large multicentre radiogenomics studies involving close co‐operation across many countries have been successfully undertaken recently. REQUITE (Validating pREdictive models and biomarkers of radiotherapy toxicity to reduce side‐effects and improve QUalITy‐of‐lifE in cancer survivors) has its own associated biobank of centrally held samples, collected regularly from local centres [39]. The REQUITE project has patient representatives on both its management and publication committees. Patient involvement with such large projects is internationally acknowledged and implemented but seldom reported in publications. However, many smaller collections lack sufficient transparency to ensure that sample access can extend beyond the immediate clinical trial group to prevent monopolisation of the resources. The question of how applications for sample access are assessed and by whom is still an issue.

## The UK radiotherapy biobanking landscape

To understand the current situation with respect to the collection of, and access to, biological samples in radiotherapy research in the UK, we devised a questionnaire that was sent to the 55 members of the NCRI Clinical and Translational Radiotherapy Research Working Groups (CTRad), requesting information regarding whether they collected tissue samples and, if so, the type of tissue samples, logistical infrastructure, current permissions regarding the sharing of tissue samples, and identifiable barriers to the storage and/or sharing of tissue samples (supplementary material, Section S1).

**Figure 1:**
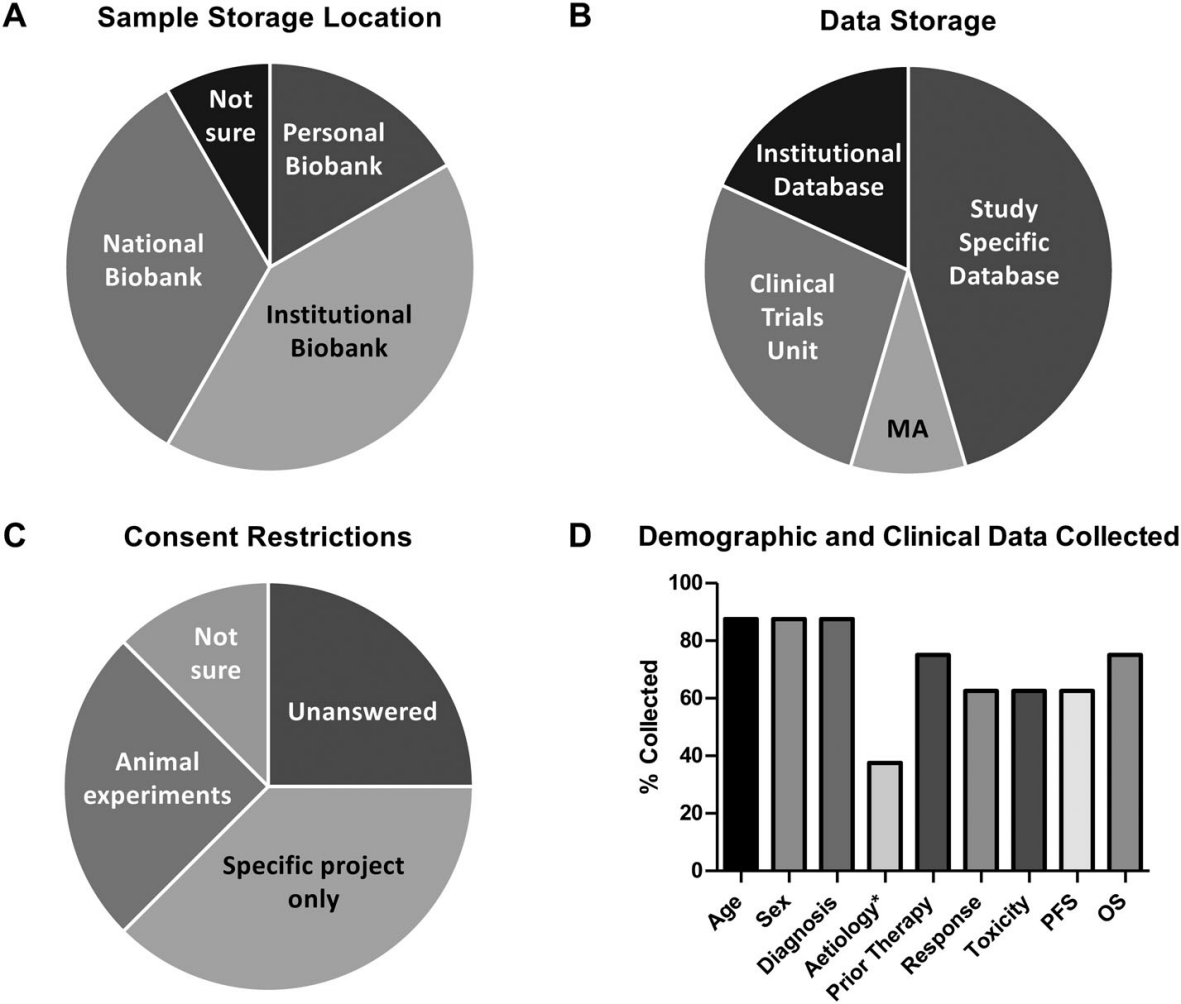
Response to CTRad survey regarding collection of samples and data. (A) Where samples are collected, (B) how the data relating to the samples are stored, (C) restrictions on consent form (animal experimentation may not be permitted at all or only with restrictions, and (D) variability in the demographic and clinical data collected. Data are from 12 respondents out of 55 solicited. *Aetiology: relevant aetiological data (e.g. smoking history, HPV, body mass index, etc). MA, medical achiever; OS, overall survival; PFS, progression‐free survival.

A total of 12 responses were obtained with 8 responders collecting tissues and 4 not collecting. The small sample size prohibited formal statistical analysis but provided a useful insight into the current landscape. Only two respondents collected tissue samples for general use with the remainder stating that consent was only obtained for sample use in the specified project, and samples could not be shared. However, most respondents would have been happy to share samples and data if access was appropriately peer reviewed and all would be happy to collect into a central biobank using a generic consent form and collection of a minimum data set if templates had been available. The majority of tissue samples and related data were stored in a study‐specific database or a clinical trials research unit that were either part of a national, institutional, or individual biobank (Figure 1A,B). One barrier to sharing was discrepancies in the level of detail given regarding the future use or sharing of collected samples on the consent form. Detail ranged from ‘donating tissue for future research’ to specifying a permitted location, team, and funding for future research (Figure 1C). There was also variation in the demographic/clinical data collected (Figure 1D).

**Figure 2:**
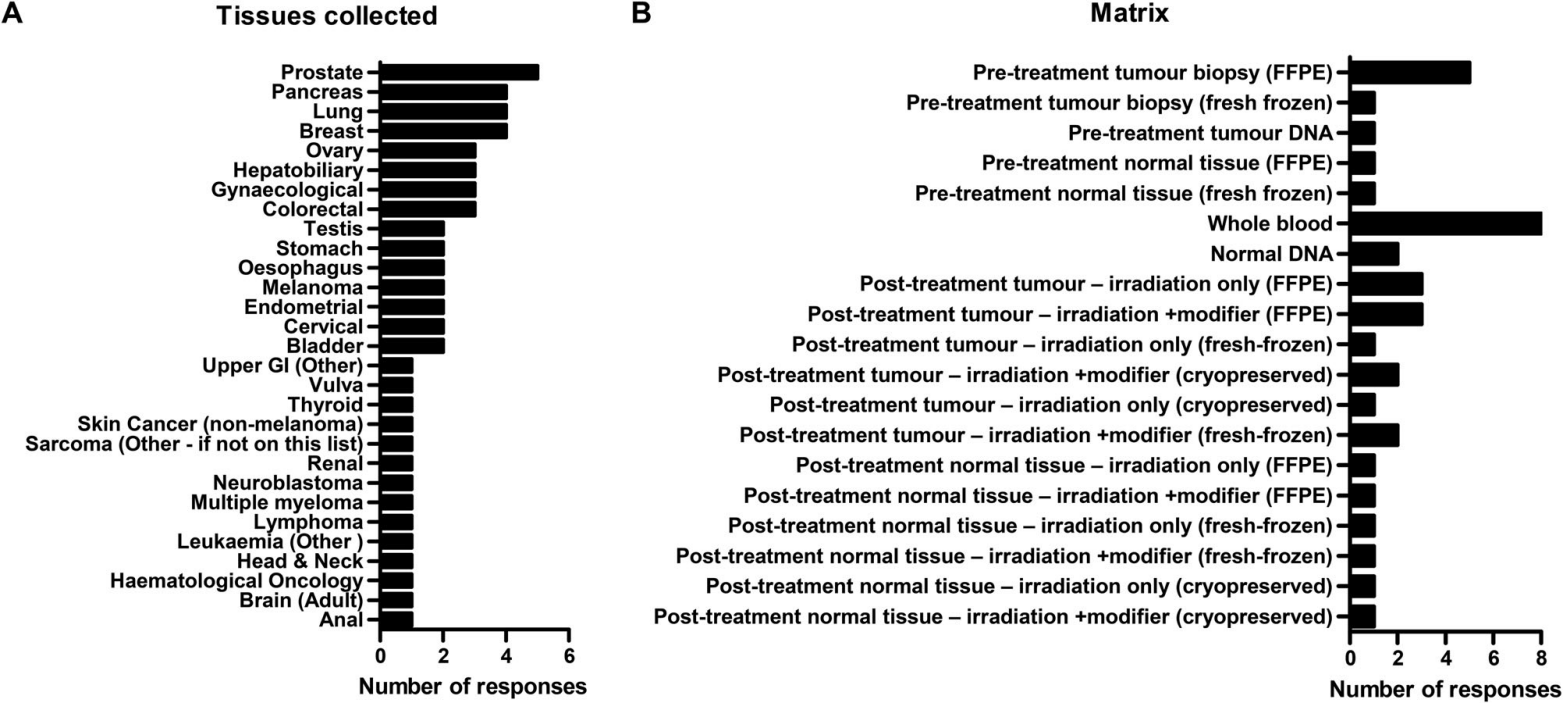
Responses from CTRad questionnaire regarding tissue collection and storage matrix. (A) Tumour types collected and (B) nature of samples taken. Data are from 12 respondents out of 55 solicited.

Numerous tumour sites were represented amongst samples collected, with the highest number of responses for samples of the prostate, pancreas, lung, and breast (Figure 2A) largely reflecting the clinical use of radiotherapy [40]. Samples were also collected at various time points in the treatment pathway (Figure 2B).

Regarding administrative support for biobanking, the most common was a data manager, followed by a biobank manager and quality assurance manager. The most common barrier to collecting, processing, and banking of tissue was a lack of facilities followed by a lack of biobanking support and lack of sample tracking software. These responses echo attitudes and practices uncovered by a related survey [23]. A second survey sent to the entire CTRad membership 2 years later elicited 57 responses of which 36 groups had the opportunity to collect patient material. Although only six answered all the questions, the data reflected the previous survey in that collections were generally as part of a clinical trial and those who did not collect cited lack of infrastructure or funding as the reason.

**Figure 3:**
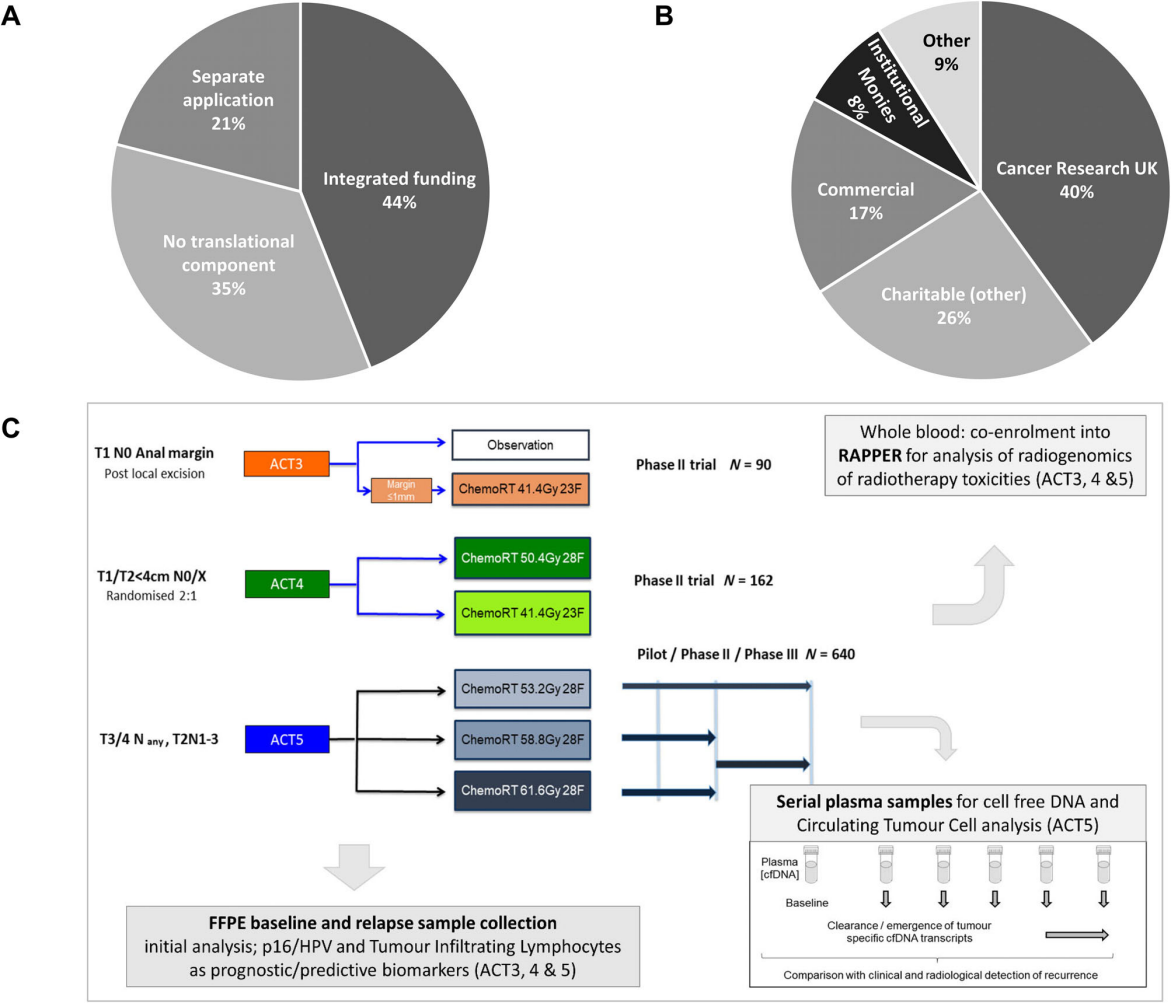
Status of tissue collection activity in recent UK radiotherapy studies. (A) Data from 57 (of 68 surveyed) UK radiotherapy studies. (B) Funding source for tissue collection for 35 studies that report biobanking biological material. (C) Integrating a sample collection [formalin‐fixed, paraffin‐embedded (FFPE) baseline and relapse samples] plus serial plasma in high‐risk cases for cell‐free DNA and CTCs within the UK Anal Cancer Platform trial PLATO (PLATO; PersonaLising Anal cancer radioTherapy dOse) maximises translational opportunities [42]. Patients were also co‐enrolled into RAPPER, obtaining a whole blood sample for genome‐wide association studies into the radiogenomics of toxicity [46]. Future radiomic and gut microbiome studies are in development.

Recognising the low response rate to our survey, we contacted the chief investigators of all (68) studies held on the NIHR Clinical Research Network (CRN) Portfolio of clinical trials (March 2019) involving radiotherapy and/or observational/biomarker studies [41]. Responses were obtained from the investigators of 57/68 (84%) radiotherapy studies. Of these, 37 trials (65%) included translational components, with 35 collecting biological material. Twenty‐five studies incorporated biobanking funding within the main study grant; the other 12 entailed separate, sequential applications (Figure 3A). The vast majority (35/37) were undertaking exploratory work – only 2 used collected biological material as stratification factors for the trial. These components were charity funded in 23 of 37 cases, many (14) by the Cancer Research UK. Six were funded by commercial (pharmaceutical industry) partners and three supported by the local academic institution. This distribution of funding sources concurs with responses to the initial questionnaire, suggesting most were funded by national or international charities (Figure 3B).

One example is the anal cancer platform PLATO (PersonaLising Anal cancer radioTherapy dOse; ISRCTN88455282) [42] funded by the Cancer Research UK and Stand Up to Cancer. Chemoradiotherapy represents standard of care in anal squamous cell carcinomas [5] where it is curative in this HPV‐associated cancer [43]. Key translational questions include those around HPV transformation, radiotherapy sensitivity/response, and prediction of radiotherapy toxicity [44]. The integrated sample collection and biomarker analysis project (Figure 3C) funded in parallel includes a serial plasma collection, addressing these questions and more. Patients enrolled in PLATO are also encouraged to provide samples within the RAPPER study (Radiogenomics: Assessment of Polymorphisms for Predicting the Effects of Radiotherapy), investigating genomic predictors of toxicity [45, 46]. With >10,000 patients, RAPPER is the largest radiogenomics resource in the world.

These data are in many ways encouraging. Nearly two‐thirds of current UK clinical trials involving radiotherapy include collection/analysis of biological samples, supported by a variety of funders. Mostly, the translational work is exploratory; we are not yet in the era of biologically stratified radiotherapy studies. In addition, it remains unclear just how accessible this material, together with the important clinical demographic and treatment details, might be to future researchers. In registering a UK clinical trial, the Integrated Research Application System [47] specifically asks what will happen to tissue samples at the end of a study, providing the option to deposit samples within a biobank and using this option would ensure tissue samples can be made available. Internationally, one such exemplar is the French glioblastoma biobank [48].

## Future perspectives

Material is clearly being collected from patients undergoing radiotherapy across a number of clinical trials but samples are neither as visible nor as accessible as they might be, use diverse consent forms, and collect different data points. With visibility and appropriate consent, they could be used by other researchers to improve radiotherapy (see the checklist in supplementary material, Section S2), for example, biomarkers predictive of (progression‐free) survival following radiotherapy [49]. Advances in understanding the role played by DNA repair mechanisms in the cellular response to ionising radiation [50], and how the tumour micro‐environment and immune response modulate outcomes [51], present a plethora of candidate biomarkers for translational research aiming to optimise radiotherapy treatments. The recent development of relatively non‐invasive liquid biopsies (ctDNA and CTCs) means there is a greater potential to collect material for research into the molecular basis of radiosensitivity and toxicity, as exemplified recently by analyses of ctDNA collected from oesophageal cancer patients [52]. However, this can only be optimally exploited if the material is made available to diverse groups. The use of such tests has already impacted clinical decision‐making in the non‐radiotherapy setting such as the plasmaMATCH trial, which successfully used ctDNA to identify breast cancer patient subgroups who were sensitive to targeted therapies [53].

To address the lack of standardisation across consent forms, and associated discrepancies in permission for sharing tissue samples between studies, a standardised consent form may be desirable (supplementary material, Table S1). This was unanimously supported by respondents to the initial questionnaire, provided that sufficient support would be made available, and is an objective of CMPath. Such consent forms must consider the patients' perspective, and recognise that cultural and spiritual backgrounds may have a bearing in this regard [54, 55]. One important issue concerns the use of samples by pharmaceutical companies and for‐profit researchers in discovery/validation programmes to avoid exploitation and promote accountability and recompense [56]. Involvement of patients as collaborators through Public Patient Involvement improves donor retention and recruitment. In addition, engagement with scientists improves the understanding of the importance and value of these gifted samples, and the expectations patients have on the output of the research.

Similarly, collection of a standardised set of clinical data (Table 1) would also facilitate sharing of sample collections and the acquisition of large numbers of samples pooled from several small studies to generate meaningful data. One issue here is the need for standardised terminology when describing available samples and the metadata associated with a sample. The Minimum Information about Biobank Data Sharing (MIABIS) approach provides a route to allow samples to be identified at the individual sample level and identification of similar samples across biobanks and networks [57]. As an extension of this core data set, consented access to extended medical information of participants would allow future enrichment of any banked tissue samples, and this data use is largely accepted by the public in the UK. The Health Data Research UK (HDR‐UK) provides a framework for acquiring consented access to medical information, providing secure data curation, and interoperable standards for data handling [58]. Similar activities such as ‘All of Us’ allow curated large‐scale secure access to health data [59]. Integrating these data through ‘Research Data Hubs’ then means that specific large‐scale data sets can be created and analysed in a secure environment with appropriate involvement of patient groups [60].

**Table 1:** Recommended data set for biobanking.

Unique identifier (e.g. collection number with abbreviation for hospital and patient's initials)	
NHS number (to allow future linkage subject to appropriate data governance)	
Consent details	Consent form number, date of consent, name of person who took it
	Consent coverage: general/specific project, consent to genetic (DNA) analysis
	Consent opt‐outs, e.g. no commercial use
	Consent active/withdrawn
	Lab number (unique identifier for anonymisation)
Demographics	Diagnosis (including stage/grade, if [when] known) and date of diagnosis
	Age at diagnosis
	Sex
	Religious or moral status
Sample details	Tissue collection date
	Sample site (whole blood/lymphocytes/tumour/lymph node/adjacent normal tissue, etc)
	Sample description note (e.g. taken × h after treatment)
	Sample type (fresh/snap frozen/cryopreserved/FFPE or derivative sample, e.g. normal/tumour DNA)
	Storage location and number of aliquots if relevant
	Available for loan
	Histology sample number
Treatment details	Prior therapy and when given, if known
	Post sample therapy planned, if known
	Radiotherapy modality, dose, fractionation, and treatment volumes
Additional treatment	Chemotherapy/endocrine therapy/targeted therapy/immunotherapy details
Outcomes	Date of first recurrence (if/when known)
	Date of death (when known and cause of death)
	QOL/patient‐reported outcomes (including late effects)

FFPE, formalin‐fixed paraffin‐embedded tissue; QOL, quality of life.

Virtual tissue banks that allow potential researchers to locate and request the use of specific samples exist, notably the UK Prostate Cancer Sample Collection Database [61]. Attached to each sample collection are data on the type of sample, location, and contact information for requesting use, for example, for the ProtecT (Prostate Testing for Cancer and Treatment) study [62]. Virtual tissue banks based on sample databases could act as models for a potential CTRad database. The UK Clinical Research Collaboration Tissue Directory (UKCRCTD) [63, 64] serves as an online centralised register of relevant tissue samples in the UK, allowing researchers to locate specific samples, with researchers then contacting individual banks directly to access samples. Similarly, BBMRI‐ERIC [65] provides a European and also international database that links national registers such as UKCRCTD to their national counterparts [66], with similar resources present in the US and Canada [67]. This provides greater access to potentially rare samples and cohorts, but also harmonisation of processes. A standardised ontology for biobanks has been derived through BBMRI ERIC [68], and the MIABIS initiative [69] describes a hierarchical approach to allow banks, samples, and data to be described, providing interoperability across banks by incorporating accepted codes (e.g. the systemised nomenclature of medicine [SNOMED]) where appropriate. Despite these initiatives, many UK cancer biobanks still lack visibility, presenting challenges for researchers in identifying available samples and how these may be accessed [70] (see the checklist in supplementary material, Section S2).

Clear information on how biobanks indicate sample access and usage, and how applications for tissue are assessed, is needed. Standardised terms of access that provide a framework for both public and researchers to understand how samples and data can be accessed have been provided by the UK NCRI and other large organisations [71, 72]. Many tissue banks employ this process with access policies built around the process of applying for tissue. These focus on the eligibility to apply, the application process itself, the review of the application, who will be involved with the review of applications, the terms and conditions of access, and how the process is governed. Individual access policies for banks should be developed with the support of patient groups who should form an integral part of the access committee.

How do biobanks indicate access and usage for the informed patient and public at large? One possibility would be a dashboard system on their website displaying metrics such as number of samples in, number of access applications made, and the number of samples released to demonstrate that samples and data are being used [20, 21]. Transparency of access is an issue but, with centralised resources such as those provided by the Medical Research Council's Regulatory Support Centre [73], the use of templates describing access terms is becoming more common, with Research Tissue Banks providing these for applicants [72]. The application process for tissue can often be seen as opaque, and there is no unified standard for what constitutes an access committee. As a key principle, donor or lay input to evaluation of any application for tissue should be valued as equal to those of the scientists running the tissue bank. This would provide donors in particular, but also potential applicants from the translational radiotherapy research community, with the assurance that tissue banks are not static collections, but dynamic structures with a specific unified aim of distributing samples and improving patient care.

## Recommendations for improvement

While good progress has been made in biobanking, there is still room for improvement in the radiotherapy field. Our data from the UK demonstrate a significant missed opportunity, given the number of patients receiving radiotherapy within clinical trials. Importantly, many biological samples and data sets potentially available (from clinical trials) will have broader relevance to the research community than the limited primary outcome measures might involve. Here, we champion some aspirational challenges for what could be done to achieve these aims for radiotherapy biobanking:

1. Due regard for the feelings, wishes, and rights of patients and participants related to: (i) transparency and rigor regarding access and research project approval; (ii) publications that acknowledge their anonymous contribution; (iii) generic consent forms and patient information leaflets that are easily understandable by patients (see supplementary material, Table S1 and Section S3); (iv) appropriate consent, curation, and storage to facilitate sharing of samples and data with the wider scientific community when the project is complete; and (v) the use of cost recovery from both academic and commercial users based on standardised tariffs for samples and data to support the long‐term operation of the biobank.
2. Standard sample collection pathways. Quality guidelines and standards exist for biobanking [27] (see also supplementary material, Table S2), but do not seem to be adopted universally by those banks that are not required to follow these regulations. National and international guidelines have also been introduced and guidance provided by, for example, BBMRI‐ERIC [64], providing frameworks that even small tissue banks can utilise. Additional professional standards for biobanking have been introduced such as ISO21899:2020 and ISBER Best Practice Guidance [74, 75], although given the constraints placed on funding, these standards may be outside the budgets of many small biobanks. Stakeholders must overcome the challenges of local, regional, and national legislation, and come together to agree a universal standard for radiotherapy biobanking.
3. To facilitate sample discovery, standard checklists for applicants should be visible on biobank websites (see supplementary material, Section S2). These should detail sample types, who can access these, how (and by whom) applications are assessed, and if there are cost recovery mechanisms [70]. In this respect, use of centralised registries such as the UKCRC platform or the MIABIS ontology provides a way of biobanks being identified and utilised.
4. Sharing resources through virtual biobanks. The NCRI Prostate Cancer Initiative mentioned earlier, or the model implemented by SEARCHBreast, provides suitable templates [76]. In these examples, tissues remain at the originating laboratory but are listed on a secure searchable online database where researchers can find, share, or upload materials. With researchers placing increased demands on biobanks, in terms of the range and type of tissues and data being requested, one of the biggest challenges faced, particularly by smaller biobanks, is operational cost. Most biobanks are poorly resourced and often struggle to cover their costs. Funders may consider cost‐sharing models whereby funding larger centralised facilities may provide economies of scale. Similarly, virtual biobanks offer a way of reducing, although not completely eliminating, such costs. In this respect, funders should consider making a condition of funding the improved visibility of biobanks.
5. Informatics allowing tissue to be linked to patient data represents a long‐term goal to identify how biomarkers are related to patient therapies and outcomes. The HDR‐UK [77] has provided a platform for health records and biomarker data to take place based on a series of specific tasks that allows for curated data analysis.

## Summary

To conclude, we champion:

- Patient involvement from the outset, facilitated by generic consent forms which are easily understandable by lay persons
- Widespread sample discoverability and availability (no ‘ownership’; collegiality, considering patient wishes)
- Easy linkage to clinical data
- Sharing data (avoiding duplication of effort)
- Standard sample collection pathways

And appeal to funding bodies to prioritise these efforts.

## Author contributions statement

NJC and VS conceived the project. GH, DCG and NJC analysed the data. GH and DCG collected the data. All authors contributed to the drafting and editing of the manuscript.

## Supporting information


**Section S1.** Questionnaires
**Section S2.** Suggested checklists to improve biobank visibility and transparency for researchers
**Section S3.** Key points for the patient information leaflet
**Table S1.** Generic consent form
**Table S2.** Existing guidelines
