# Supplementary material for: Radiotherapy biobanking: current landscape, opportunities, challenges, and future aspirations
Source: J Pathol Clin Res. 2021 Oct 17;8(1):3–13. doi: 10.1002/cjp2.246 (PMC8682944; doi:10.1002/cjp2.246)
Supplement: Supplementary file 1 — Section S1. Questionnaires Section S2. Suggested checklists to improve biobank visibility and transparency for researchers Section S3. Key points for the patient information leaflet Table S1. Generic consent form Table S2. Existing guidelines [file CJP2-8-3-s001.pdf]

Radiotherapy biobanking: current landscape, opportunities, challenges, and future aspirations

TH Ward *et al*. *J Pathol Clin Res* DOI: 10.1002/cjp2.246

Supplementary Material

Section S1. Questionnaires

Section S2. Suggested checklists to improve biobank visibility and transparency for researchers

Section S3. Key points for the patient information leaflet

Table S1: Generic consent form

Table S2. Existing guidelines

Section S1. Questionnaires

A. Initial survey to CTRAD WS1 and WS2 (2016)

1. **Do you collect material from patients or healthy volunteers?** Y/N (if No please go to question 13)
2. **If yes, are the samples** (tick all that apply):
    a. Collected for a defined research project which has current valid ethical approval
    b. Collected/stored for a clinical trial which has current valid ethical approval
    c. Collected and stored as part of a Human Tissue Authority Research Tissue Bank (see Q6)
    d. Other (please specify)
3. **Is your sample collection funded** (tick all that apply)?
    a. By a Research Council
    b. By a National/International Charity
    c. Commercially
    d. Internal funding
    e. No current funding
4. **Have you discussed the collection of material with anyone in the CTRad Biomarker panel of experts?** Y/N
5. **What tissues do you collect** (tick all that apply)
    a. Pre-treatment tumour biopsy (FFPE)
    b. Pre-treatment tumour biopsy (fresh frozen)
    c. Pre-treatment tumour biopsy (cryopreserved)
    d. Pre-treatment tumour DNA
    e. Pre-treatment normal tissue (FFPE)
    f. Pre-treatment normal tissue (fresh frozen)
    g. Pre-treatment normal tissue (cryopreserved)
    h. Whole blood
    i. Normal DNA
    j. Post-treatment tumour – irradiation only (FFPE)
    k. Post-treatment tumour – irradiation +modifier (FFPE)
    l. Post-treatment tumour – irradiation only (fresh-frozen)
    m. Post-treatment tumour – irradiation +modifier (cryopreserved)
    n. Post-treatment tumour – irradiation only (cryopreserved)
    o. Post-treatment tumour – irradiation +modifier (fresh-frozen)
    p. Post-treatment normal tissue – irradiation only (FFPE)
    q. Post-treatment normal tissue – irradiation +modifier (FFPE)
    r. Post-treatment normal tissue – irradiation only (fresh-frozen)
    s. Post-treatment normal tissue – irradiation +modifier (fresh-frozen)
    t. Post-treatment normal tissue – irradiation only (cryopreserved)
    u. Post-treatment normal tissue – irradiation +modifier (cryopreserved)
    v. Other (please specify)
6. **Are they collected into a biobank** (tick applicable, may be >1)?
    a. Your own
    b. Departmental
    c. Institutional
    d. A biobank that is part of the Confederation of Cancer Biobanks
    e. Not sure

7. **Who takes consent?**
    a. Clinician
    b. Research or Tissue Bank nurse
    c. Not sure
    d. Other (please specify)
8. **Does the consent form have restrictions regarding the use of the material** (tick all that apply)?
    a. For a specified project only
    b. Animal experimentation
    c. Commercial use
    d. UK only
    e. Not sure
    f. Other (please specify)
9. **What clinical details do you record as part of the biobanking?** (tick all that apply)
    a. Age
    b. Sex
    c. Diagnosis
    d. Relevant etiological data (e.g. smoking history, BMI, HPV)
    e. Prior therapy
    f. Response
    g. Toxicity
    h. Progression-free survival
    i. Overall survival
    j. Not sure
    k. Other (please specify)
10. **How is the data stored?**
    a. Study-specific database
    b. Medical Achiever
    c. Clinical Trials Research Unit
    d. Not sure
    e. Other (please specify)
11. **What support do you have for Biobanking** (tick all that apply and give FTE)?
    a. Biobank manager
    b. Quality assurance manager
    c. Data manager
    d. Not sure
    e. Other (please specify)
12. **Would you be happy/permitted to share the samples and/or data with other scientists/clinicians that could lead to a larger study?**
    a. Samples Y/N
    b. Data Y/N
    c. Not sure
13. **If you answered No to Q1 are there any barriers preventing you from banking material or processing samples** (tick all that apply)?
    a. Lack of facilities
    b. Lack of biobanking staff support
    c. Lack of sample tracking software
    d. Not sure

e. Other (please specify)

14. **Would you consider collecting material into a CTRad (or joint CTRad/CMPath) biobank using a generic consent form and collection of a minimum dataset regarding patient demographics and clinical details, if support was given?** Y/N

B. Second Survey to CTRad membership (2019):

1. Are you involved in clinical radiotherapy where patients samples are collected or there is the opportunity to collect them? Y/N If Y go to questions 4 and 5

2. Are you involved in pre-clinical radiotherapy where samples are collected or there is the opportunity to collect them? Y/N

3. Do you work on in vitro models of radiotherapy Y/N

4. 1. If you don't currently collect samples can you tell us what are the barriers to collection (e.g. lack of funding, infrastructure/personnel support/HTA compliance etc.)?

OR

5. If you do collect/have collected samples can you tell us:

   a) What tumour type (e.g. lung, breast, all-comers etc)?
   b) What type of sample (e.g. whole blood, germline DNA, tumour: snap frozen/FFPE/DNA etc.)?
   c) Are the samples pre- or post radiotherapy or both?
   d) Is the collection part of a clinical trial?
   e) Do you have permission to share the samples and would you be willing to?
   f) Roughly how many samples do you have?

C. Survey to principal investigators (PIs) conducting Clinical trials:

1. Is there translational work currently funded?
2. Is this for biological material or imaging biomarkers (or both)?
3. Who is it funded by?
4. Was it funded as part of the main grant or as a subsequent application?
5. Is it used to stratify treatment (within the trial) or exploratory?

Section S2. Suggested checklist to improve visibility and transparency of biobanks for researchers (adapted from [45])

- Website
- Types of samples available
- Access process and any restrictions to access e.g. to commercial organisations
- Timeline for assessing applications
- Cost recovery
- Ethics statement
- Contact details for the biobank (email and/or telephone)

Section S3. Key points to include in the Patient Information Leaflet

Generic Patient Information Leaflet

Several generic Patient Information leaflets exist:

https://www.ukbiobank.ac.uk/media/ei3bagfb/participant_information_leaflet-baseline.pdf
print-ready-version-nov-2017-v10-blue-infosheet.pdf (nibiobank.org)
Patient Information Sheet | Wales Cancer Bank

Similarly, the Health Research Authority and Medical Research Council Regulatory Support Centre have provided guidance on producing a Patient Information Leaflet that is clear and understandable. We recommend using these as a guideline and such leaflets should encompass the following key aspects to mirror what the participant will be providing consent for in the Consent Form (see Table S1): Genetics, Ethics, Toxicity, PROMS, funding of the biobank, for-profit organisations such as pharmaceutical companies, development of cell cultures from tissues including stem cells, use of samples in animals, development of diagnostic tests and Quality of Life (QOL) data. In developing a Patient Information Leaflet, there is ample opportunity for patients to help to shape such leaflets to make them both understandable and also to provide input on any potentially contentious issues. A simple set of areas to be covered in a generic leaflet is described below.

1. Invite the patient to take part explaining how important their samples are and how they will be stored for future use in a biobank. Explain that the research may not specifically benefit them but could be used to help other people with similar conditions in the future.
2. Explain what a Radiotherapy biobank is (a big store cupboard/freezer) and how it works, including who funds it and how it is regulated by a specific Access Committee. Include information about how radiotherapy patients like themselves are involved in the running of the biobank in matters of regulation and ethics. State that they will not benefit financially from this, but travel expenses may be claimed.
3. Say why they have been chosen; for example, 'You are being asked to donate some of your tissue, that is in excess of that needed for your diagnosis or treatment, for research because you are going to have some radiotherapy. Patients differ in how radiotherapy works on them, both in terms of effectiveness and side-effects. The reason for these differences is not

completely understood. Research is needed to find out the causes of these differences so that treatment of patients in the future can be improved'.

4. State clearly that their taking part is voluntary and that they may change their mind at any time, and this will not affect their current or future treatment. Samples and or data collected thus far will be retained or destroyed depending on their wishes.
5. Emphasise that their information will be kept confidential and anonymised and it won't be possible for researchers to identify who they are.
6. Reassure the participants by letting them know that any information stored about them will always be anonymous and that governments, insurance companies, private healthcare providers, or employers will not have access to this data.
7. State what will happen to them during the process of giving a sample. This may be routine biopsy/surgery/blood tests or additional tests separate from their usual care. Let them know what the experience is likely to be.
8. Describe the advantages of contributing to the biobank and indicate the scope of the research their samples may be involved in. Let them know how long their samples and data could be kept for i.e. ten years or more. Indicate that their samples and data could be used for research well into the future, and might involve their samples and data being used in techniques that we cannot predict. The research might also help other research being carried out by other scientists in approved centres. Such applications will be carefully vetted by the biobank management team including patient representatives who would be able to provide a patient perspective on any research.
9. Emphasise that what happens to the samples and data will be determined entirely by the participant, that they can decide what happens with their samples and that this can be as broad as possible or can be restricted according to their views (tiered consent).
10. Let the participant know that commercial organisations such as pharmaceutical companies might want to use their samples for their own research. Indicate what the potential benefits of this might be such as newer drugs becoming available in the future or new ways to diagnose patients but that companies will naturally aim to get back their costs.
11. Explain that the bank may charge researchers, universities or organisations a suitable fee for providing the samples and that this is to help maintain the bank and their samples and data. Make it clear that the charge to researchers will not be for the samples themselves, but for the upkeep of the storage facilities and for the people who are actually involved in banking and distributing samples and data.
12. Explain that their genetic material (DNA) will be collected and used and that such samples may be copied (possibly repeatedly), stored, and distributed, to ethically approved organisations. Discuss what the implications of this might be for them and possibly their families.
13. Indicate why animals are sometimes needed for research and what the samples could be used for, which types of animals would be used, and how this type of research will be carefully regulated. Emphasise that using their samples in this type of research will always be according to their wishes.
14. Explain what will happen to their samples if they are no longer considered useful for research and the reasons why the samples might not be useful, such as simply not enough sample left. Indicate what would happen to samples if for any reason the biobank must close.

Table S1. Generic Consent Form: minimum items to include, can be tiered/expanded to take account of religious/ethical sensitivities [78].

**Name and Identifier[1]:**	**Please Initial**
I have read and understood the patient information leaflet and been able to consult others (family, carer, GP) and to ask questions.	…………
I know who to contact should I have any additional questions about my samples and data.	…………
I understand my participation is voluntary and I am free to withdraw from sample donation at any time without giving a reason and this will not affect my current or future treatment (medical care).	…………
I understand I will not receive results of any tests or research carried out on my samples unless these tests may influence my treatment or affect the health of my family (genetic research).	…………
I understand that the bank may operate "cost recovery" to maintain the bank and my samples and data (information).	…………
I consent to the donation (gifting) of samples collected from me in the past and present that are no longer needed for diagnosis or treatment.	…………
I consent to my samples being shared in an anonymised form to approved researchers for ethically approved medical research.	…………
I consent to my data (information) being stored and shared in an anonymised form to approved researchers electronically for ethically approved medical research.	…………
I consent to my medical notes and any questionnaires to be looked at by responsible persons associated with the banking of my samples and those individuals involved in research relating to my condition or participation in a clinical trial as long as my personal details remain confidential.	…………
I consent to sharing data (information) and samples with other approved institutions and organisations in other countries for the purpose of this and future research.	…………
I consent to my samples being used for research and development associated with my condition by commercial organisations.	…………
I consent to my data (information) and medical records in an anonymised form being accessed by commercial organisations involved in research and development.	…………
I understand that I may be re-contacted by the Biobank (e.g. to answer some more questions and/or attend another assessment visit), but this will be optional	…………
I understand that my identity will be anonymised (kept confidential) and there will be no intention or capability to trace research back to me (the donor/patient).	…………
I consent to my genetic material being collected and that such samples may be copied, stored, and distributed, to ethically approved organisations.	…………
I consent to my tissues being used to develop cell cultures and these cell cultures may be grown indefinitely, stored, and distributed, to ethically approved organisations.	…………
I consent to my tissue being grown in animals[2] (state species) under appropriate national standards for ethically approved research.	…………
I understand I will not personally receive any financial payment for my samples at any time, but the bank may charge a suitable fee for samples to help maintain the bank and my samples and data.	…………
I consent for my samples to be used for the development of novel tests and the certifying of such tests for medical use.	…………
I understand that when my samples are no longer of use for research, they may be used for training purposes or destroyed according to approved standards.	…………

To provide the widest use of biobanked samples and data, participants should be provided with the opportunity to give broad and enduring consent. Templates and guidance for these approaches are

provided by certain key organisations such as the Health Research Authority (HRA) and MRC Regulatory Support Centre and these can be adapted to suit specific needs including those for paediatric donors:

http://www.hra-decisiontools.org.uk/consent/index.html

We outline certain key items that participants and families should be allowed to consider when donating samples and data. Specific items that are potentially contentious to participants will depend on their outlook, but should include genetic analysis, the derivation of cell lines, use of samples and data by commercial organisations, and cost recovery to aid with running biobanks. In these circumstances, participants should have the option to decide which of these items they wish to consent to separately, rather than simple broad consent or no consent.

[1]All consent forms should declare the name and academic affiliation of the bank, the name of the study (or clinical trial where appropriate), the ethics approval number including version number, the trial/study patient identifier, the patient NHS number or similar unique linked identifier to allow future linkage subject to appropriate data governance. Most consent forms require patients to initialise each statement rather than tick Yes/No. It should be stated that the form has been approved by a Research Ethics Committee, passed through quality control and has been validated, followed by a QA signature or stamp.

[2]One sensitive issue is the use of animals in research studies, and there is the opportunity to allow a participant varying degrees of involvement from no use of animals, to using only certain species such as rats or mice, according to their own views.

There are a number of UK national biobanks that have similar consent forms:

https://www.ukbiobank.ac.uk/media/t22hbo35/consent-form.pdf
http://www.nibiobank.org/for-patients
https://www.walescancerbank.com/patient-consent-form.htm

Table S2. Existing guidelines of relevance to biobanking

Guideline	Description	Reference
SPREC (Standard PREanalytical Code)	Identify and control pre-analytical variables during tissue collection which may influence quality	[79]
BRISQ (Biospecimen Reporting for Improved Study Quality)	Tiered system which evolved from SPREC to provide further standardisation and consistency for tissue collection	[80]
CMPath Biobanking Sample Quality Improvement Tool	Tissue collection self-assessment tool with guidance on how to increase the quality of tissues collected	[24]
CTRNet (Canadian Tissue Repository Network)	Set of operational standards, which all biobanks in the network must follow to gain accreditation	[81]
Human Tissue Authority Codes of Practice	Guidelines for taking consent and the operation of research tissue banks to HTA standards	[82]
IARC/WHO Standards	Helpful Standard Operating Procedures for the IARC Biobank	[83]
GDPR and Biobanking Individual Rights, Public Interest and Research Regulation across Europe.	A BBMRI-ERIC: (Biobanking and BioMolecular resources Research Infrastructure - European Research Infrastructure Consortium) initiative which gives a pan European perspective on national legislation relative to biobanking across EU Member States. The book is open access.	[84]
